# Relationship between Stress and Neuroimmunological Responses and Health Literacy in Newly Diagnosed HIV-Infected Patients: An Exploratory Study

**DOI:** 10.1155/2024/3432569

**Published:** 2024-09-20

**Authors:** Bengt B. Arnetz, Judith E. Arnetz, Norbert Kaminski, Ryan Tomlin, Andrew Cole, Pamela Bartlett, Robert Crawford, Andrew Jameson

**Affiliations:** ^1^ Department of Family Medicine Michigan State University College of Human Medicine, Grand Rapids, Michigan, USA; ^2^ Department of Pharmacology and Toxicology Michigan State University College of Human Medicine, East Lansing, Michigan, USA; ^3^ Institute for Integrative Toxicology Michigan State University, East Lansing, MI, USA; ^4^ Department of Pharmacy Trinity Health Grand Rapids Hospital, Grand Rapids, Michigan, USA; ^5^ Department of Research & Innovation Trinity Health Grand Rapids Hospital, Grand Rapids, Michigan, USA; ^6^ Department of Medicine Trinity Health Grand Rapids Hospital, Grand Rapids, Michigan, USA; ^7^ Department of Medicine College of Human Medicine Michigan State University, East Lansing, MI, USA

## Abstract

**Objectives:**

We aimed to study self-rated health and psycho-neuroimmunological responses during the initial 6 months after testing positive for human immunodeficiency virus (HIV) and its relationship to health literacy, that is, the ability to take in and understand information about one's illness. Health literacy plays a critical role in patients' ability to adhere to antiretroviral treatment (ART). However, there is a lack of studies on the possible impact of HIV-induced proinflammatory processes on health literacy.

**Methods:**

Twelve patients with newly diagnosed HIV attending an urban Ryan White-funded HIV clinic responded to a questionnaire and had blood samples drawn at baseline (first visit) and after 1, 3, and 6 months, respectively. The questionnaire measured stress, depression, and health literacy. Blood was analyzed for HIV RNA plasma viral load, CD4 cell count, pro- and antistress, and inflammatory markers.

**Results:**

Complete data for the entire 4 collection periods were available for nine patients. Over the 6-month period, mean viral load decreased from 353,714.83 (standard deviation 870,334.61) to 35.89 (14.04) copies/mL (*p* < 0.001). CD4 cell count increased from 321.08 (167.96) to 592.44 (300.06) cells/mm^3^ (*p* < 0.001). Self-rated stress decreased from a baseline mean of 7.33 (2.29) to 3.56 (3.21), on a 0–10 visual analogue scale, at the 6-month follow-up (*p* < 0.01). C-reactive protein (CRP) decreased from 5757.05 (3146.86) to 2360.84 (2277.33) ng/mL (*p* < 0.05). Mean health literacy score at baseline was 17.67 (3.50; scale range 0–20) and did not change during the follow-up period. However, increased stress and decreased CRP (*p* = 0.05) during the 6-month follow-up predicted higher health literacy scores at 6 months.

**Conclusion:**

Both stress and proinflammatory processes in newly diagnosed HIV-infected patients might adversely impact patients' health literacy and thus their capacity to align with treatment guidance.

## 1. Introduction

Infection by the human immunodeficiency virus (HIV) is associated with strong psychological and neuroimmunological responses [1]. The systemic immune system activation is reflected in increased levels of circulatory soluble inflammatory cytokines and reduced CD4 blood cell count [2]. The neuroimmunological response contributes to patients commonly experiencing intense and sustained psychological stress that can negatively impact both mental health and cognitive functioning [3, 4].

HIV-associated increases in circulatory cytokines and immune cells initiate microglial proliferation in several key brain structures, including the hypothalamus. The local inflammation is believed to cause synaptic remodeling and neurodegeneration within the hypothalamus, interfering with hypothalamic outputs contributing to cognitive impairment mediated by hippocampus, amygdala, and reward-processing centers [5]. The syndrome is referred to as HIV-associated neurocognitive disorders (HAND) and can occur in HIV-positive persons, even when there is no detectable circulating HIV due to effective antiretroviral treatment (ART) [6]. The cognitive deficits appear close to the initial infection and prior to ART [7, 8]. The mechanisms behind HAND are not fully known [9]. In addition to the infection per se, cerebral small vessel disease (CSVD) has been discussed as a possible contributing factor [6]. During the early phase of the disease, asymptomatic neurocognitive disorders and mild cognitive disorders are more common [10]. Thus, HIV, stress, inflammation, and cognitive impairment are closely related. However, the temporal and causal relationships between these mechanisms are still not well understood [10, 11].

It is well-documented that health literacy, that is, the ability to comprehend disease-specific information [12, 13], is an important determinant of the ability to align with treatment protocols and with HIV disease outcomes [6, 14]. Disease-specific health literacy is a very focused domain, and it might be that a person living with HIV (PLWH) ability to process complex medical information, in a commonly stressful context, might be affected prior to changes in neuropsychiatric test performance.

Less is known about the possible impact of HIV-induced psycho-neuroimmunological processes on health literacy. What is the relationship among neuroimmunological processes, patients' psychological and medical trajectories, and health literacy during the initial phase of living with HIV [6, 15]?

Stress and poor mental health in PLWH are associated with increased health risk behaviors, decreased quality of life and treatment adherence, and accelerated disease progression [1, 16, 17]. The psycho-neuroimmunological responses during the initial phase of HIV are often substantial and complexly interrelated [18]. Psychological stress attenuates neuroimmunological defense processes [19, 20]. Furthermore, sustained stress has been associated with worse psychological adjustment reflected in increased depression and anxiety symptoms in PLWH [4].

In summary, the literature documents substantial stress, depression, and neuroimmunological responses in persons with newly diagnosed HIV infection [1, 3, 4]. However, although neuroimmunological responses apparently normalized during the first year of effective treatment, a large proportion of patients on ART continue to report that the “most bothersome symptoms were related to mental health and central nervous system” even after being on ART for a decade or more with implications for health-related quality of life and ART discontinuation [17]. To better understand the interaction among neuroimmunological processes and self-reported health, well-being, and cognitive function during the early phase of the HIV infection, we need studies that include such a multidisciplinary approach and that data are collected more frequently than what is currently the case [8, 10, 15, 21, 22]. For example, Cole et al. [8] used several methodologies, including neuroimaging and neuropsychological assessments, to determine changes in PLWH versus controls. However, assessments were done only twice, at baseline and after 2 years. Moreover, the possible relationship between neurocognitive and neuroimaging parameters and participants' health literacy capacity was not assessed. Boerwinkle et al. [10] summarized more recent studies concerning the temporal relationship between PLWH and structural and neurocognitive changes. The authors conclude that “HIV triggers strong neuroimmune response and may lead to a cascade of events including increased chronic inflammation and cognitive decline” [10]. Aung et al. [15] reviewed cognitive health in PLWH. They specifically addressed neuroinflammation, depressed mood, and immune activation during the first 12 months of HIV infection. They addressed the need for data to better understand HIV-triggered neurocognitive and inflammatory changes during the early phase and their reversibility with initiation of early ART. The current study adds to the current understanding how HIV triggers immune, neurocognitive, and mood changes by focusing more specifically on the first 6 months after HIV acquisition, as well as relating HIV-triggered inflammation to perceived stress, depressed mood, and health literacy. Such studies are likely to contribute new knowledge that would benefit both HIV theory and clinical practice.

In the current pilot project, we studied self-reported stress and depression and concentration of neuroimmunological markers in blood and their relationship to health literacy in newly HIV-diagnosed patients during the initial 6 months.

## 2. Materials and Methods

### 2.1. Survey Design and Participants

This was a prospective pilot study, in which 12 individuals with newly diagnosed HIV were followed for 6 months. Responses to self-reported health and well-being items, further detailed below, and blood samples were collected a total of four times, that is, at the initial visit to the HIV clinic (baseline), and after 1, 3, and 6 months, respectively.

Complete data were secured from nine individuals.

### 2.2. Variables

During each of the four visits to the clinic, patients responded to a questionnaire consisting of eight items, of which the following 4 items were used in the current study: How stressed are you right now, How often do you feel depressed, How confident are you filling out medical forms by yourself, and How often do you have someone help you read hospital materials? Responses were provided on a visual analogue scale (VAS) ranging from 0 to 10. The stress and depression items have been validated in prior studies [23–25].

Health literacy was assessed by two validated items—Confidence filling out medical forms and Frequency getting help reading medical materials (reversed scoring), from the Newest Vital Sign Scale [26]. The item sum score was used to generate the Health Literacy Scale. The minimum score was 0 points, and maximum was 20 (highest possible literacy).

Self-reported stress was assessed using the one-item visual analogue scale (VAS): “How stressed are you right now.” Responses were provided on a visual analogue scale (VAS) ranging from 0 to 10, validated in prior studies [23–25].

Levels of HIV and CD4 were determined on whole blood samples using the Abbott Alinity m platform and BD FACSLyric, respectively. Remaining whole blood samples were processed on-site, lysing and stabilizing solutions added, and refrigerated at +4 degrees Centigrade for 2 weeks and then transferred to −80 degrees Centigrade.

Whole blood samples were allowed to clot by leaving it undisturbed at room temperature for 20 minutes and then centrifuged at 2,000 g for 10 minutes. The resultant supernatant, designated serum, was extracted and frozen at minus 80 degrees Centigrade after aliquoting for later analysis. Serum was analyzed for the following inflammatory markers, in a BSL 2+ security-classified laboratory: interferon-ϒ (IFN-*γ*), interleukin-1*β* (IL-1*β*), interleukin-2 (IL-2), interleukin-6 (IL-6), interleukin-8 (IL-8), interleukin-18 (IL-18), tumor necrosis factor alpha (TNF-*α*), and C-reactive protein (CRP). Serum levels of interleukin-10 (IL-10) were used as an indicator of anti-inflammatory processes. Serum levels of the hormone cortisol were used as a marker of catabolism/stress. Anabolic or antistress activity was determined using serum levels of the hormone dehydroepiandrosterone-sulfate (DHEA-s). The ratio between catabolic and anabolic neuroendocrine activity was calculated by the formula cortisol/DHEA-s.

Serum levels of IFN-*γ*, IL-1*β*, IL-2, IL-6, IL-8, IL-18, IL-2, IL-8, TNF-*α*, IL-10, and cortisol were quantified using a customized LEGENDplex panel (Biolegend, San Diego, CA) per manufacturer's protocol. Samples were evaluated on a Cytek Northern Lights flow cytometer and analyzed using the Data Analysis Software Suite by Biolegend. Serum DHEA-s was quantified by competitive ELISA (Invitrogen, Frederick, MD) and serum CRP by ELISA (Invitrogen) per manufacturer's protocol. Both ELISAs were quantified using a BioTek Synergy plate reader (Agilent, Santa Clare, CA).

### 2.3. Selection and Recruitment

Participants were recruited among individuals with newly diagnosed HIV-positive status that had been referred to an urban, Ryan White-funded HIV clinic in the Midwest United States.

The baseline data were collected between October 2021 and February 2023. Six-month follow-up data were consequently collected between March 2022 and August 2023. The healthcare providers, including a Doctor of Pharmacology, were specialists employed by the clinic and responsible for caring for the participating people living with HIV.

Written informed consent was obtained from all participants.

### 2.4. Sample Size

This was a pilot study to determine the feasibility of the study design and to secure the collection of hypothesis-generating preliminary data focusing on the relationship among stress, neuroimmunology, and health literacy. We had no specific a priori hypothesis, and we did not conduct a formal statistical power analysis. The current study was deemed sufficient to generate data for sample size analysis for a larger, future study.

### 2.5. Statistical Analysis

Statistical analysis was done using the statistical software package IBM SPSS, version 29. Following review of mean and dispersion measures, chi-square statistics were used to test for possible differences across discrete variables. Nonparametric and Student's *t*-tests were used to test for differences in continuous variables across groups. Paired sample *t*-tests were used to compare changes between baseline and the 6-month follow-up specifically. One-way analysis of variance was used to test for possible differences over the entire study period. Biological variables were transformed using natural logarithm (Ln) when not normally distributed. Linear regressions were used to test whether self-reported stress, depression, stress and antistress hormones, and inflammatory biomarkers predicted health literacy scores at the 6-month follow-up assessment, controlling for baseline health literacy scores. Statistical significance was set at a two-sided *p* value <0.05.

## 3. Results

The median time between having tested positive for the HIV and the baseline visit to the HIV clinic was 20.5 days (25^th^ percentile 9.5 days, 75^th^ percentile 30.25). Eleven participants self-identified as men and one as woman. Seven (58%) were White, of which 2 were Hispanic, 3 (25%) were African Americans, and 2 (17%) chose not to report race/ethnicity. The mean age was 37.0 years old (S.D. 12.5), with a range from 24 to 68 years. Two participants self-identified as straight, 1 as bisexual, 5 as gay or lesbian, and 4 chose not to respond to the question. Three patients missed one or more appointments resulting in 9 patients being followed with complete data for the initial 6 months.

### 3.1. Changes in Individual Self-Reported Health and Biomarkers at Baseline and 1, 3, and 6 Months

Table 1 reports data for the 9 participants that had attended all four data collection points. The median self-rated stress decreased statistically significantly from 7.00 at baseline to 2.00 at the 6-month follow-up, and blood cell counts of HIV decreased significantly from a median of 20,306.00 to 40.00, while CD4 cell count in blood increased significantly from a median of 238.00 to 552.00.

In terms of immunological markers, there were no significant changes using a one-way analysis of variance including all four assessment times in serum CRP, IFN-ϒ, IL-1*β*, IL-2, IL-6, IL-8, IL-10, IL-18, and TNF-*α*. There were also no significant changes over time in the catabolic/stress hormone cortisol, the anabolic/antistress hormone dehydroepiandrosterone-sulfate (DHEA-s), nor in the ratio between cortisol and DHEA-s.

Self-reported depression and health literacy did not change during the 6-month period.

Table 2 depicts that, while stress decreased significantly over the study period for the participants as a group, there were noticeable interindividual differences in the stress response. The coefficient of variance was the lowest at baseline (6.33), then increased during the initial 3 months of treatment (12.75) and continued to be elevated even at the 6-month follow-up (10.28). In contrast, the coefficient in Ln CRP fluctuated during the initial 6 months from a baseline of 2.10 ng/mL to 1.45 at 1 month, 2.49 at 3 months, and 1.10 at 6 months.

### 3.2. Changes between Baseline and the 6-Month Follow-Up

We were also interested in determining whether there were any statistically significant changes between baseline and the 6-month follow-up assessment, using paired samples *t*-tests and Wilcoxon signed-rank tests. We had complete data for ten out of the twelve participants since the analysis required that the participant had at least participated in baseline and 6-month follow-up assessments. Self-reported stress decreased significantly between baseline and the 6-month follow-up. The mean decrease in stress was 3.77 (S.D. 2.43; standardized test statistics *W*_9_ = −2.68, two-sided *p* value = 0.01). The mean decrease in depression scores was 0.78 (1.20), which was statistically nonsignificant (*W*_9_ = 2.50, two-sided *p* value = 0.08). There were no statistical changes in the health literacy scale or in the two individual scale items.

In terms of changes in biological variables, the analysis showed a significant decrease in viral load from baseline to the 6-month follow-up, with a mean decrease of 450,221.67 copies/mL (998,598.20; *W*_9_ = 2.67, *p* < 0.01), while CD4 T cells increased by a mean of 260.78 cells/mm^3^ (186.34; *W*_9_ = 2.67, *p*=0.01).

Looking specifically at the inflammatory and neuroendocrine markers, including the ratio between serum cortisol and DHEA-s, the only statistically significant change was a mean decrease in CRP of 2549.26 ng/mL (2246.96; *W*_7_ = −2.03, *p*=0.04). IL-10, an anti-inflammatory marker, decreased a mean of 3.83 pg/mL (9.62, *W*_7_ = −2.02, *p*=0.04, test on Ln-transformed values). For all other biomarkers, there were no significant changes between baseline and the 6-month follow-up.

## 4. Predictors of Changes in Health Literacy Scores

Self-reported stress and depression, and inflammatory biomarkers were used as independent variables in a linear regression to predict health literacy scores at the 6-month follow-up, controlling for baseline health literacy scores.  Table 3 shows that changes between increased stress and decreased inflammatory drive, as measured using CRP (*p*=0.05), between baseline and 6 months predicted higher health literacy scores at 6 months.

## 5. Discussion

The focus of this study was to determine whether self-reported stress and a select set of neuroimmunological measures changed between baseline and the 6-month follow-up in newly diagnosed people living with HIV. Moreover, we were interested in whether changes in stress and neuroimmunological biomarkers were prospectively related to changes in health literacy scores during the initial 6 months after HIV diagnosis.

As expected based on prior work, viral load decreased significantly between baseline and the 6-month follow-up, while CD4 T cells increased significantly during the same period [2]. Self-rated stress decreased significantly during the study period, while depression ratings remained stable, as did health literacy scores. Prior work has reported similar findings for depression [22]. However, less attention has been paid to possible changes in self-perceived stress during the initial phase. Importantly, there were noticeable interindividual differences in most self-reported as well as biological stress markers. This points to the importance of further studies to better understand the basis for the variations in self-reported and neuroimmunological biomarkers. Based on the coefficient of variance, the interindividual variance in self-reported stress was the lowest at baseline to increase during the initial treatment period.

CRP, an important clinical marker of systemic inflammation, decreased significantly when comparing baseline to 6-month follow-up values [27–29]. The decrease in CRP reflects the well-known overall decrease in systemic inflammatory drive following the initiation of antiretroviral therapy (ART) [30–32]. However, when all 4 assessment periods were included in a one-way ANOVA, the change over time did not reach statistical significance. This might be due to a rather gradual decrease in CRP over time. Alternatively, that the study sample was too small. Future studies need to determine whether there is a lag in anti-inflammatory processes following ART that requires a more extensive follow-up period before it is statistically significant. CRP did not exhibit the same large interindividual variation as was observed for self-rated stress. IL-10, an anti-inflammatory and proenergy marker, showed a limited, but statistically significant, decrease when comparing baseline to 6-month follow-up [33]. This might indicate that the IL-10, rather than increasing over time to counter the proinflammatory effects of CRP, decreases thus furthering system-level inflammation. CRP is a rather nonspecific marker of systemic inflammation that is commonly used clinically and is reflective of numerous upstream inflammatory processes that were not captured in our limited set of other proinflammatory markers. The lack of significant decreases in these other inflammatory markers might also reflect a time delay between ART-induced viral suppression and a decrease in specific inflammatory processes driven by interleukins and interferons [2].

Although several of the study variables showed significant changes between baseline and the 6-month follow-up, only changes in self-rated stress, viral load, and CD4 counts were found to be statistically significant when repeated-measures analysis was applied including all 4 time points from baseline to 6 months, with appropriate control for repeated measures. This suggests that changes over the 6-month period are rather slow, and more frequent data collection over a longer time is warranted.

As hypothesized, decreased inflammation, measured by serum CRP, was marginally but significantly predictive of higher health literacy scores at 6 months. In contrast to many other studies linking HIV-induced neuroimmunological processes to cognitive functions, we did not use resource-requiring neuropsychiatric tests [8]. Rather, we based our assessment on a patient-centered health literacy survey about how PLWH's comprehension of medical information might have changed over time. Decreased CRP levels predicted increased health literacy scores at 6 months. This finding supports the notion that HIV-induced systemic inflammation might impact cognitive function, and earlier in the disease phase, than has been recognized in prior work using neuropsychiatric tests [2, 5, 11]. This adds to previous research reporting a cognitive decline in virally suppressed PLWH over a longer time, suggesting that neurocognitive impairment might occur earlier than expected in the disease process, possibly prior to changes in traditional neuropsychiatric tests [14, 34]. Prior work has shown that suboptimal adherence to ART therapy is associated with increased system inflammation although the patients' viral load in blood is suppressed [30, 31]. However, these authors did not consider that increased system inflammation, not suboptimal adherence to ART, could be the causative mechanism.

Increased stress from baseline to the 6-month follow-up predicted higher health literacy scores, controlling for baseline values. It could be related to a recovery of an initially fatigued neuroendocrine stress response capacity. However, this finding needs to be studied further in future, with larger samples.

We used both pro- and anti-inflammatory interleukins to determine possible effects on health literacy. The only significant marker, as discussed above, was CRP, which is an unspecific marker of downstream systemic inflammation. Moreover, interleukin-10, an established anti-inflammatory and proenergy marker, was not associated with health literacy [33].

### 5.1. Limitations

This was a pilot study of a purposefully selected small group of PLWH that recently had been diagnosed with an HIV infection. Since we worked with a marginalized group of people, it is a challenge to retain them in a longitudinal study. However, thanks to the HIV clinic's commitment to support patients from all walks of life, the retention rate was high. Although a small study, we monitored self-reported and biomarker-based health indicators rather frequently during the first 6 months, and our loss to follow-up was limited. The limited number of participants and the fact that all participants were recruited from one, midwestern HIV clinic, limits the generalizability of the study. However, the repeated data collection over a rather short time and the focus on the initial 6 months following diagnosis adds important data to the extant literature.

## 6. Conclusion

Both stress and proinflammatory processes in newly diagnosed HIV-infected patients might adversely impact patients' health literacy and thus their capacity to align with treatment guidance. Results highlight the importance of more detailed studies of the initial phase after acquiring HIV in terms of psychological and neuroimmunological responses. Moreover, there is a need to enhance our understanding of how early psycho-neuroendocrine changes, along with the viral load, might impact a PLWH's health literacy and ability to comprehend and adhere to treatment protocols.

## Figures and Tables

**Table 1:** Changes in individual self-reported health and biomarkers at baseline and 1, 3, and 6 months.

Category	Baseline	1 month	3 months	6 months	Statistics
Self-ratings	Median (25, 75%)	Median (25, 75%)	Median (25, 75%)	Median (25, 75%)	
Stress (0–10)	7.00 (5.00, 9.00)	6.00 (0.00, 7.00)	2.00 (0.00, 7.00)	2.00 (0.00, 7.00)	*F*_df=3_=11.50*p*=0.02
Depression (0–10)	4.00 (2.00, 9.00)	5.00 (2.00, 8.00)	8.00 (0.00, 9.00)	5.00 (0.00, 9.00)	*F*_df=3_ = 1.27 *p*=0.31
Confidence (0–10)	10.00 (8.00, 10.00)	10.00 (8.00, 10.00)	10.00 (10.00, 10.00)	10.00 (10.00, 10.00)	*F*_df=3_ = 1.22 *p*=0.33
Help (0–10)	0.00 (0.00, 3.00)	3.00 (0.00, 5.00)	6.00 (0.00, 7.00)	2.00 (0.00, 6.00)	*F*_df=3_ = 1.56 *p*=0.23
Health literacy scale	19.50 (16.25, 20.00)	18.00 (13.00, 20.00)	20.00 (12.50, 20.00)	18.00 (13.50, 20.00)	*F*_df=3_ = 1.24 *p*=0.32
HIV status					
(Ln)^1^ viral load (copies/mL)	20,306.00 (4246.00, 148313.00)	40.00 (40.00, 107.00)	40.00 (35.00, 40.00)	40.00 (20.00, 40.00)	*F*_df=3_ = 9.01, *p*=0.02
CD4^1^ count (cells/mm^3^)	238.00 (143.00, 444.00)	533.00 (307.00, 559.00)	506.00 (379, 718.00)	552.00 (379.00, 667.00)	*F*_df=3_ = 57.75, *p* < 0.001
Biomarkers					
IFNy^1^ (pg/ml)	1.10 (1.10, 34.43)	1.10 (1.10, 88.30)	1.10 (1.10, 1.15)	1.10 (1.10, 40.03)	*F*_df=3_ = 1.37 *p*=0.29
IL-1B^1^ (pg/ml)	7.20 (7.20, 24.43)	7.20 (7.20, 49.79)	7.20 (7.20, 7.20)	7.20 (7.20, 32.13)	*F*_df=3_ = 1.00 *p*=0.42
IL-2^1^ (pg/ml)	0.68 (0.68, 10.29)	0.68 (0.68, 39.48)	0.68 (0.68, 0.91)	0.68 (0.68, 13.40)	*F*_df=3_ = 1.06 *p*=0.394
IL-6^1^ (pg/ml)	1.61 (1.61, 36.19)	1.61 (1.61, 277.11)	1.61 (1.61, 2.03)	1.61 (1.61, 29.04)	*F*_df=3_ = 1.94 *p*=0.17
IL-8^1^ (pg/ml)	1.80 (1.80, 15.01)	1.80 (1.80, 41.18)	1.80 (1.80, 1.91)	1.80 (1.80, 7.74)	*F*_df=3_ = 1.00 *p*=0.42
IL-10^1^ (pg/ml)	0.55 (0.48, 17.08)	0.56 (0.48, 57.34)	0.49 (0.48, 0.67)	0.48 (0.48, 19.85)	*F*_df=3_ = 1.50 *p*=0.25
IL-18^1^ (pg/ml)	153.79 (18.55, 512.58)	148.57 (35.35, 372.33)	120.98 (41.34, 147.39)	127.73 (71.22, 222.70)	*F*_df=3_ = 0.56 *p*=0.652
CRP^1^ (ng/ml)	4965.52 (3176.72, 7783.41)	2142.24 (671.34, 7842.67)	2056.03 (1355.60, 5331.90)	1969.82 (741.38, 3931.03)	*F*_df=3_ = 0.42 *p*=0.74
TNF − *α*^1^ (pg/ml)	0.68 (0.68, 39.47)	0.68 (0.68, 186.74)	0.68 (0.68, 63.85)	0.68 (0.68, 0.68)	*F*_df=3_ = 1.45 *p*=0.27
Cortisol^1^ (ng/ml)	85.23 (33.78, 617.94)	82.44 (32.68, 284.62)	190.67 (55.89, 275.17)	105.62 (31.93, 206.76)	*F*_df=3_ = 0.34 *p*=0.80
DHEA-s^1^ (ng/ml)	3351.14 (987.88, 6248.71)	3201.42 (340.57, 6711.50)	3400.32 (1028.71, 5787.90)	3972.84 (1626.74, 7601.96)	*F*_df=3_=0.85*p*=0.49
Cortisol/DHEA-s	0.58 (0.54, 0.60)	0.58 (0.53, 0.65)	0.58 (0.49, 0.67)	0.59 (0.47, 0.70)	*F*_df=3_=0.46*p*=0.72

^1^Ln = transformed using natural logarithm. Statistics: one-way analysis of variance.

**Table 2:** Group median and variance and individual changes in self-rated stress during the initial 6 months following HIV diagnosis.

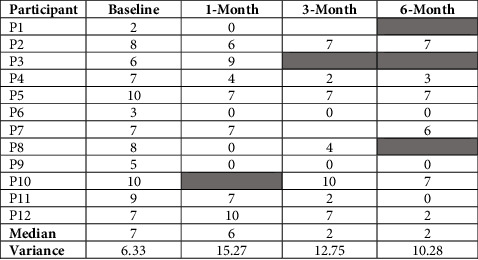

Shaded areas represent sampling episodes in individual participants who did not participate in.

**Table 3:** Self-reported and biological predictors of health literacy scores at 6 months controlling for baseline health literacy scores.

Dependent variable: Health literacy scores at 6 months					
Model	Stand. Β	*t*	Sig	95% conf. interval	
				Lower	Upper
(Constant)	0.35^1^	0.54	0.65	−2.44	3.14
Health literacy scale at baseline^2^	1.47	20.63	0.002	0.97	1.49
Baseline (Ln)CRP minus (Ln) CRP at 6 months	0.15	4.22	0.05	−0.01	1.13
Baseline stress minus stress at 6 months	−0.59	−8.26	0.01	−2.2	−0.69

Adj *R* square 0.99, SE 0.31, *R* square change 0.99, *F*_df 2_ = 269.97, *p*=0.004, Durbin–Watson 2.84. ^1^Unstandardized, ^2^Health literacy score is the sum score of items—confidence filling out medical forms and frequency getting help reading medical materials (reversed scoring), from the Newest Vital Sign Scale [26].

## Data Availability

The dataset and related data dictionary are available from the corresponding author on reasonable requests.
